# Supplementary material for: Artificial 2D van der Waals Synapse Devices via Interfacial Engineering for Neuromorphic Systems
Source: Nanomaterials (Basel). 2020 Jan 2;10(1):88. doi: 10.3390/nano10010088 (PMC7022853; doi:10.3390/nano10010088)
Supplement: Supplementary file 1 [file nanomaterials-10-00088-s001.pdf]

Supplementary Information

Artificial 2D van der Waals Synapse Devices via Interfacial Engineering for Neuromorphic Systems

Woojin Park[1], Hye Yeon Jang[1], Jae Hyeon Nam[1], Jung-Dae Kwon[2] and Byungjin Cho[1*], Yonghun Kim[2*]

[1] Department of Advanced Material Engineering, Chungbuk National University, Chungdae-ro 1, Seowon-Gu, Cheongju, Chungbuk, 28644, Republic of Korea; wjpark@chungbuk.ac.kr (W.P.); hyjang0581@gmail.com (H.Y.J.); jhnam0714@gmail.com (J.N.); bjcho@chungbuk.ac.kr (B.C.)

[2] Materials Center for Energy Convergence, Surface Technology Division, Korea Institute of Materials Science (KIMS), 797 Changwondaero, Sungsan-gu, Changwon, Gyeongnam, 51508, Republic of Korea; jdkwon@kims.re.kr (J.D.K); kyhun09@kims.re.kr (Y.K.)

[*] Co-correspondence: bjcho@chungbuk.ac.kr (B.C.); Tel.: +82-(0)43-261-2417, kyhun09@kims.re.kr (Y.K.); Tel.: +82-(0)55-280-3281 Received: date; Accepted: date; Published: date

Keywords: 2D heterostructure; WSe_2; $NbSe_2$; Nb_2O_5 interlayer; synapse device; neuromorphic system

1. Raman spectrum of WSe_2 and $NbSe_2$ after transfer process

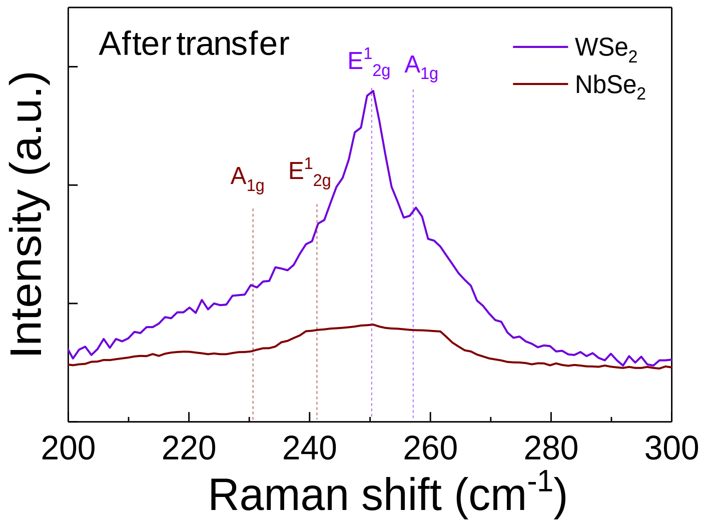

Figure S1. Raman spectrum of WSe_2 and $NbSe_2$ with stacked device structure after transfer process

2. Repeatability test of DC transfer double sweep curves with different Nb_2O_5 thicknesses

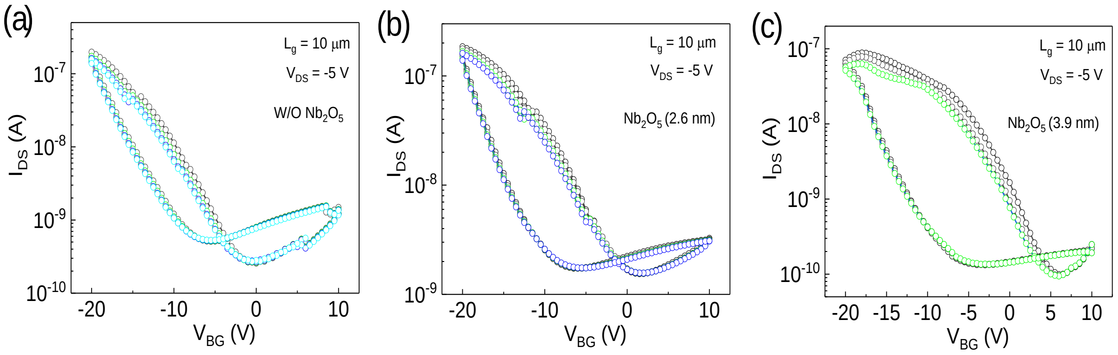

Figure S2. Repeating test of DC transfer curves with different Nb_2O_5 thicknesses

3. The dependence of DC sweep speed on transfer curves

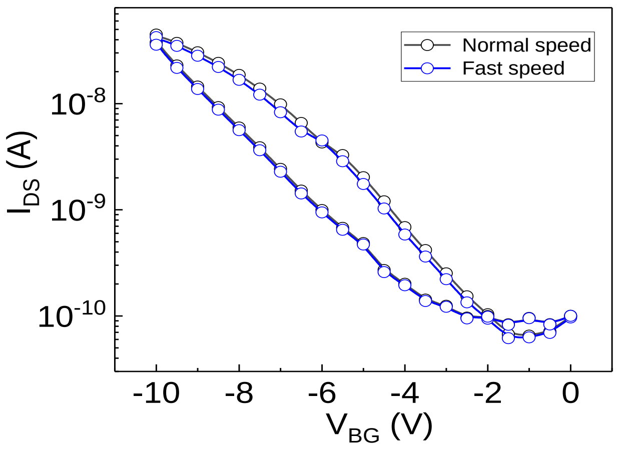

Figure S3. The dependence of DC sweep speed on transfer curves with 2.6 nm thickness of Nb_2O_5
